# Trends in HPV‐associated cancer incidence in Texas medically underserved regions

**DOI:** 10.1002/cam4.70133

**Published:** 2024-08-27

**Authors:** Thao N. Hoang, Abbey B. Berenson, Yong Shan, Fangjian Guo, Victor Adekanmbi, Christine Hsu, Xiaoying Yu, Yong‐Fang Kuo

**Affiliations:** ^1^ Institute for Translational Sciences University of Texas Medical Branch Galveston Texas USA; ^2^ Center for Interdisciplinary Research in Women's Health University of Texas Medical Branch Galveston Texas USA; ^3^ Department of Obstetrics and Gynecology The University of Texas Medical Branch at Galveston Galveston Texas USA; ^4^ Department of Biostatistics and Data Science University of Texas Medical Branch Galveston Texas USA

**Keywords:** disparities, epidemiology, human papillomavirus

## Abstract

**Background:**

While cervical cancer incidence rates (IR) in the United States have dropped in the last 20 years, non‐cervical human papillomavirus (HPV) associated cancers increased. Many people in Texas (TX) live in medically underserved areas and have higher risk of developing HPV‐associated cancers. Since previous studies of these regions focused on cervical cancer, we included other HPV‐associated cancers in our analysis of IR in East TX and the TX‐Mexico Border compared to other TX regions.

**Methods:**

Cancer data from 2006 to 2019 were obtained from the TX Cancer Registry. Cases of HPV‐associated cervical, vaginal, vulvar, penile, anal, and oropharyngeal cancers and corresponding patient‐level demographic data were included. We calculated IR per 100,000 and drew heat maps to visualize cancer IR by county. To control potential confounders, we added county‐level risk factors: rates for smoking, excessive drinking, obesity, STIs, primary care provider availability and dentist availability, from the County Health Rankings and Roadmaps program. We reported IRs by region and time and estimated unadjusted and adjusted risk ratio (RR) for association of each type of cancer and region. Lastly, we created adjusted models for each cancer by period to see time trends of regional differences.

**Results:**

Risk of anal, cervical, and oropharyngeal cancer was lower at parts of the Border than in the rest of TX in the adjusted model. We also observed increasing anal and oropharyngeal cancer risk and decreasing cervical and vaginal cancer risk over time.

**Conclusion:**

Patient sociodemographics, behavioral risk factors, and access to care may contribute to some observed differences in cancer IR across regions. This indicates that targeted prevention efforts towards these regions, especially in low socioeconomic status communities, may benefit future generations.

## 1 INTRODUCTION

Human papillomavirus (HPV) is the most common sexually transmitted infection (STI) in the United States (US).^1^ While most HPV infections are cleared naturally by a cell‐mediated immune response, some infections persist and can lead to precancerous lesions and later, cancer.^2^ HPV infection is associated with cervical, anal, oropharyngeal, penile, vaginal, and vulvar cancers.^3^ Peak HPV acquisition has been shown to occur in adolescence and early adulthood.^4^ However, due to the time course of cancer progression, peak cancer diagnosis does not occur until 45–60 years of age for cervical cancer, and the mid‐to‐late 60s for non‐cervical cancers.^4^, ^5^, ^6^, ^7^, ^8^, ^9^

The incidence rate (IR) of HPV‐associated cancers combined has increased in the past two decades, with a reported annual percentage change (APC) of 0.5% between 1999 and 2015.^10^ However, the incidence rate for cervical cancer during this time has decreased by 1.6% per year,^10^ indicating that the increased combined IR of HPV‐associated cancers can be attributed to non‐cervical cancers. This is consistent with other studies of non‐cervical cancer IRs. For example, oropharyngeal cancer IR has increased such that it is now the most common HPV‐associated cancer in the US, overtaking cervical cancer.^10^

Within Texas (TX), cervical cancer rates in the Rio‐Grande Valley (RGV) are 25% higher than in the rest of the state, and 55% higher than the US average.^11^ The RGV, which lies on the southernmost tip of the Texas–Mexico Border, is a medically underserved area, and women in the region experience a disproportionate burden of cervical cancer due to lack of access to screening services and treatment. Another medically underserved population of interest is East Texas. According to a 2021 report from The University of Texas Tyler Health Science Center, East Texans experienced higher rates of both cancer incidence and mortality than Texas overall.^12^ While prior studies have extensively studied cervical cancer rates in Texas, there is a gap in research regarding non‐cervical cancers (anal, oropharyngeal, vaginal, vulvar, and penile).^13^, ^14^, ^15^, ^16^ We therefore aimed to investigate rates of HPV‐associated cancers, including but not limited to cervical cancer in Texas, and to determine how different regions of Texas compared. We included cervical cancer in our analyses to assess its difference from non‐cervical cancers for regional comparison, and to further examine the impact of additional risk factors. We also studied trends over time by region and evaluated the association of both sociodemographic and behavioral risk factors.

## 2 MATERIALS AND METHODS

### 2.1 Data sources

We combined data from three different sources: the Texas Cancer Registry, the American Community Survey, and the County Health Rankings and Roadmaps Program. De‐identified patient‐level cancer data from 2006 to 2019 were obtained from the Texas Cancer Registry. Cases of HPV‐associated cancer are defined by International Classification of Diseases for Oncology, 3rd Edition (ICD‐O‐3) codes for sites: cervix, vagina, vulva, penis, anus, oropharynx. Sites were refined by histology codes likely to be HPV‐associated (cervical: 8010–8671 and 8940–8941, other sites: 8050–8084 and 8120–8131). To obtain the denominator population, we used data from the US Census Bureau American Community Survey (ACS), which includes county‐level sociodemographic information (population sizes by age groups, sex, and race/ethnicity). Additionally, we used corresponding data from the County Health Rankings and Roadmaps program to capture county‐level behavioral risk factors.

### 2.2 Measures

The primary outcome of interest was IR of new HPV‐associated cancer cases per 100,000 persons. Risk ratios comparing the effects of living in different regions, as well as for different risk factors, were assessed. Sociodemographic variables included age (20–44, 45–59, and ≥60), sex (male, female), diagnosis year, race/ethnicity (Hispanic, non‐Hispanic (NH) White, NH Black, NH American Indian or Alaska Native (AIAN), NH Asian or Pacific Islander (API)), poverty (percent living below poverty level), and county of residence. Other risk factors included percentage of adult smokers, percentage of adults who report binge or heavy drinking (excessive drinking), obesity (percentage of adults who report BMI ≥30), and chlamydia incidence per 100,000 to represent STI transmission at each county. We also included information on percentage of population uninsured, ratio of population to primary care physicians (PCP ratio), and ratio of population to dentists (dentist ratio) at each county to approximate access to care. Missing values in the County Health Rankings and Roadmaps data were replaced with the next available ones. For example, a missing value in the 2010 file would be replaced with one from the 2015 file. For rates of smoking and excessive drinking, about two‐thirds of counties had missing values in 2010 and 2015 and were replaced with 2019's more complete data. Since ACS provides 5‐year estimates, we divided the cases into three periods: 2006–2010, 2011–2015, 2016–2019, using ACS estimates for 2010, 2015, and 2019 respectively for each period.

Counties were sorted into groups representing our regions of interest (North Border, Central Border, South Border, East Texas, and rest of Texas). Border counties were the 32 Texas counties defined in the La Paz Agreement of 1983 as part of the Texas‐Mexico Border.^17^ To better represent the variation of healthcare needs in this large area, we then subdivided the Border region into three subregions corresponding to Texas Public Health Regions (PHR) for the regression models (North, Central, and South Borders). The decision to divide the Border into 3 regions was based on results from the Texas Department of State Health Services (DSHS) Workforce Supply and Demand Projections, which estimated different levels of unmet physician demand in different regions of this area.^18^ For rarer cancers (vaginal, vulvar, and penile), we only presented results for the combined Border region because the total number of cases was less than 11 for some subregions. The East Texas region was defined as the 38 counties in PHR 4 and 5 combined, and the “Other” region encompassed the remainder of the Texas counties not included in the previously defined regions.

### 2.3 Statistical analysis

Baseline descriptive characteristics were calculated to describe the entire population of Texas as a whole, as well as for each region for comparison. Each county was represented by six rows (representing sex percentage breakdowns for each age group) at each of the three time periods. Multiplied by the 254 counties in TX, this made for a total of 4572 total records in the aggregated analytical file. For cancers specific to males or females (cervical, vaginal, vulvar, and penile), there were 2286 records. We calculated age‐adjusted incidence rates (IR) for each HPV‐associated cancer by region, as well as by time period. We then created zero‐inflated Poisson regression models for each type of cancer to show unadjusted difference of cancer incidence, stratifying by region, using the “Other” region as the reference value. The model was offset by the log of population size. Data were checked for overdispersion and, if present, we used a zero‐inflated negative binomial model instead of a zero‐inflated Poisson model. We also created heat maps plotting our calculated IRs in counties to visualize the county‐level geographical distribution of HPV‐associated cancer risk in Texas. Subsequently, we generated full, adjusted models to assess the association between region and cancer incidence, adjusting for county‐level risk factors as potential confounders. We then created adjusted models for each cancer, stratified by year periods to observe whether regional differences change over time. Due to the limitations regarding missing data in our smoking and drinking rates, we additionally performed a second time trend analysis excluding these affected variables. This allowed us to observe the impact of our method of replacing missing values with data from the next available period. We further tested whether this difference was significant by adding the interaction between region and time period in the adjusted model including the entire period. Associations were estimated with risk ratios (RR) and 95% confidence intervals (CI). Analyses were conducted using SAS system version 9.4 for Windows (SAS Institute Inc., Cary, North Carolina) and ArcGIS. Associations were considered statistically significant with a *p*‐value < 0.05, and tests were two‐sided.

## 3 RESULTS

Differences in sociodemographic and behavioral characteristics by region are summarized in Table 1. This was calculated using 2019 data, representing the denominator population that was used to calculate the most recent period of 2016–2019. Most counties were in the rest of Texas (“Other”), so this region reflected the characteristics of Texas overall. The total population size of the Border and East TX regions were comparable (1,843,053 vs. 1,426,842). Slightly more people living in East TX were 60 years and older compared to the rest of Texas (32.2% vs. 24.0%). There were significant differences in race and ethnicity composition between the regions, with the Border region being predominantly Hispanic compared to East TX and the rest of Texas (87.9% vs. 15.7% vs. 35.4%). In contrast, East TX had the greatest proportion of non‐Hispanic White persons compared to the Border and the rest of Texas (64.2% vs. 9.3% vs. 44.2%) as well as the highest proportion of non‐Hispanic Blacks (16.7% vs. 1.3% vs. 12.6%). Compared to the other regions, counties in the Border region had the highest proportions of poverty (22.6%), uninsured individuals (23.0%), and chlamydia (432.7 per 100,000). Counties in East TX had the highest proportions for smoking (16.9%) and obesity (32.3%).

**TABLE 1:** Characteristics of study denominator population by region, 2019.

	Texas total	Regions		
		Border	East TX	Other
Characteristics	*n* (%)	*n* (%)	*n* (%)	*n* (%)
Population ≥20 years old	20,128,205 (100)	1,843,053 (100)	1,426,842 (100)	16,858,310 (100)
Sex				
Male	9,883,311 (49.1)	894,715 (48.5)	707,235 (49.6)	8,281,361 (49.1)
Female	10,244,904 (50.9)	948,339 (51.5)	719,608 (50.4)	8,576,957 (50.9)
Age group				
20–44	9,972,575 (49.5)	947,067 (51.4)	598,748 (42.0)	8,426,760 (50.0)
45–59	5,197,680 (25.8)	443,744 (24.1)	369,232 (25.9)	4,384,704 (26.0)
60+	4,957,992 (24.6)	452,226 (24.5)	458,890 (32.2)	4,046,876 (24.0)
Race and ethnicity				
Non‐Hispanic White	8,538,532 (42.4)	171,276 (9.3)	916,399 (64.2)	7,450,857 (44.2)
Non‐Hispanic Black	2,378,730 (11.8)	23,315 (1.3)	238,468 (16.7)	2,116,946 (12.6)
Non‐Hispanic AIAN	52,987 (0.3)	3468 (0.2)	5475 (0.4)	44,044 (0.3)
Non‐Hispanic API	973,734 (4.8)	17,252 (0.9)	18,447 (1.3)	938,035 (5.6)
Hispanic	7,815,531 (38.8)	1,620,312 (87.9)	224,603 (15.7)	5,970,616 (35.4)
% Poverty, mean (SD)	15.7 (6.2)	22.6 (9.7)*	17.2 (3.5)*	14.2 (4.7)*
% Smokers, mean (SD)	16.1 (1.7)	16.2 (2.1)	16.9 (1.2)*	15.3 (1.5)*
% Obesity, mean (SD)	30.4 (2.4)	29.3 (1.6)*	32.3 (2.7)*	29.7 (2.1)
% Binge drinking, mean (SD)	17.2 (1.8)	15.7 (1.8)*	17.5 (1.4)*	18.4 (1.7)*
% Uninsured, mean (SD)	20.8 (3.9)	23.0 (4.3)*	19.4 (2.6)*	19.9 (3.9)*
Chlamydia rate, mean (SD)^a^	417.4 (290.2)	432.7 (278.1)	404.4 (160.2)	415.1 (316.4)
PCP ratio, mean (SD)	36.4 (25.6)	31.0 (21.4)	34.7 (22.0)	43.5 (26.4)
Dentist ratio, mean (SD)	28.4 (21.1)	19.6 (15.8)*	33.6 (17.8)	32.1 (22.0)*

^a^ Chlamydia rate per 100,000.

* Significant difference from TX Total at *p* < 0.05 using one‐sample *t*‐test.

**FIGURE 1:**
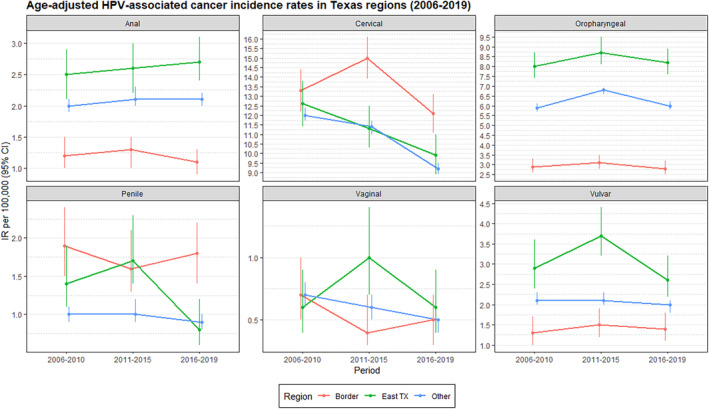
Graph of age‐adjusted HPV‐associated cancer IRs by region and period.

**FIGURE 2:**
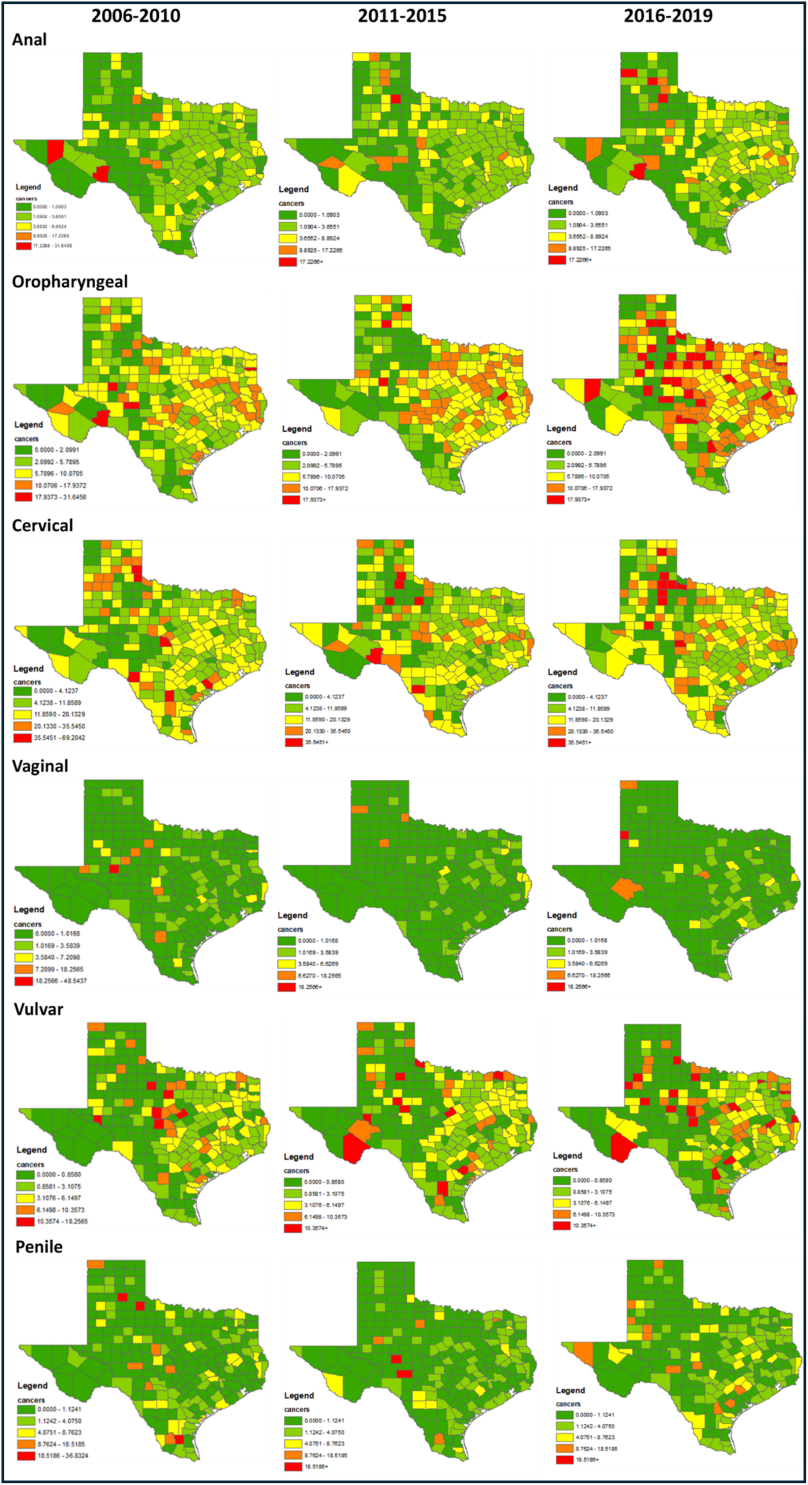
Heat map of HPV‐associated cancer incidence rates by Texas county and period, 2006–2019. Cutoff for levels (min/20th percentile/40th/80th/max) was based on the first period (2010) for each cancer and kept consistent.

Age‐adjusted IRs of HPV‐associated cancers by region and period are shown in Figure 1. Table S1 includes all numerical values for Figure 1. The heat maps generated based on county‐level IRs are shown in Figure 2. From 2006 to 2019, IR trends varied by region. Anal cancer IRs increased in East TX (2.5 [95% CI: 2.1–2.9] to 2.7 [2.4–3.1]) but were stable at the Border and the rest of TX. Oropharyngeal cancer IRs increased in East TX (8.0 [7.4–8.7] to 8.2 [7.6–8.9]) but remained stable in other regions. Vaginal cancer IRs decreased at the Border (0.7 [0.5–1.0] to 0.5 [0.3–0.7]) and the rest of TX (0.7 [0.6–0.8] to 0.5 [0.4–0.5]) but were stable in East TX. Vulvar cancer IRs increased at the Border (1.3 [1.0–1.7] to 1.4 [1.1–1.8]) but decreased in East TX (2.9 [2.4–3.6] to 2.6 [2.2–3.2]) and the rest of TX (2.1 [2.0–2.3] to 2.0 [1.8–2.1]). Cervical cancer IRs decreased less at the Border (13.3 [12.2–14.4] to 12.1 [11.1–13.1]) than in East TX (12.6 [11.4–13.8] to 9.9 [8.9–11.0]) and the rest of TX (12.0 [11.7–12.4] to 9.2 [8.9–9.5]). Penile cancer IRs in East TX declined most (1.4 [1.1–1.9] to 0.8 [0.6–1.2]) compared to other regions, which remained relatively stable.

Results from the unadjusted regression models can be seen in Table 2. In the unadjusted models, we found that risk of anal cancer was lower than in the rest of TX for persons living in the Central Border (RR: 0.38, [95% CI: 0.22–0.64]) and in the Southern Border (0.56, [0.42–0.75]). Similarly, risk of oropharyngeal cancer was lower in all Border subregions than the rest of TX. Risk of oropharyngeal cancer (1.15, [1.01–1.31]) was higher in East TX than in the rest of TX. Risk of cervical cancer was higher in the Central Border (1.44, [1.20–1.73]) and the Southern Border (1.33, [1.19–1.49]) than in the rest of TX. We did not observe any regional differences in vaginal cancer incidence. Persons living at the Border were at a lower risk of vulvar cancer (0.63, [0.48–0.84]), but had a much higher risk of penile cancer (1.87, [1.38–2.55]) than those in the rest of TX.

**TABLE 2:** Risk of HPV‐associated cancer by region, unadjusted (2006–2019).

Cancer type/region	RR	95% CI	*p*‐value
Anal			
Border (North)	0.77	0.51–1.18	0.233
Border (Central)	0.38*	0.22–0.64	0.0003
Border (South)	0.56*	0.42–0.75	0.0001
East TX	1.09	0.95–1.25	0.239
Other	REF		
Oropharyngeal			
Border (North)	0.48*	0.34–0.70	0.0001
Border (Central)	0.46*	0.32–0.66	<0.0001
Border (South)	0.46*	0.35–0.62	<0.0001
East TX	1.15*	1.01–1.31	0.038
Other	REF		
Cervical			
Border (North)	1.08	0.91–1.15	0.388
Border (Central)	1.44*	1.20–1.73	0.0001
Border (South)	1.33*	1.19–1.49	<0.0001
East TX	1.06	0.99–1.15	0.115
Other	REF		
Vaginal			
Border	0.87	0.58–1.31	0.511
East TX	1.10	0.81–1.50	0.542
Other	REF		
Vulvar			
Border	0.63*	0.48–0.84	0.001
East TX	1.14	0.95–1.37	0.163
Other	REF		
Penile			
Border	1.87*	1.38–2.55	<0.0001
East TX	1.08	0.84–1.40	0.550
Other	REF		

* *p* < 0.05.

Results from the full, adjusted models are in Table 3. After adjustment, we found that risk of anal and oropharyngeal cancer was now lower in all Border subregions than in the rest of TX. We no longer observed an increased risk of oropharyngeal cancer in East TX. Interestingly, risk of cervical cancer in the Northern Border and Southern Border were now lower than in the rest of TX. Like in the unadjusted model, we still did not observe any regional differences in vaginal cancer risk. The lower risk of vulvar cancer at the Border was also still present. However, we no longer observed an increased risk of penile cancer in the Border region. Regarding changes over time, we found that people were more likely to be diagnosed with oropharyngeal cancer between 2011 and 2015 than 2006 and 2010 (1.14 [1.08–1.19]). There was still a positive association seen with the 2016–2019 period compared to 2006–2010, but the RR was slightly lower than that of the 2011–2015 period (1.09, [1.02–1.17]). For anal cancer patients, we found increased risk of diagnosis in 2016–2019 compared to 2006–2010 (1.22, [1.09–1.37]). For cervical cancer, we saw an overall decreasing trend, with a lower risk in 2011–2015 compared to 2006–2010 (0.91, [0.87–0.96]), and an even lower risk in 2016–2019 (0.85, [0.79–0.91]). There was also a decrease in vaginal cancer diagnosis over time, with a lower risk in 2016–2019 versus 2006–2010 (0.68, [0.49–0.94]). We did not observe any significant trends over time in vulvar and penile cancer patients. Although the regional difference for cervical cancer across period decreased over time; we did not observe a similar decrease for non‐cervical cancer (Tables S2–S7). We also found negligible differences in our secondary time trend analysis when excluding smoking and drinking variables (Tables S8–S13).

**TABLE 3:** Risk of HPV‐associated cancer, adjusted (2006–2019).

Variable	Anal		Oropharyngeal		Cervical		Vaginal^a^		Vulvar		Penile	
	RR	95% CI	RR	95% CI	RR	95% CI	RR	95% CI	RR	95% CI	RR	95% CI
Sex												
Male	0.63*	0.59–0.66	5.23*	5.03–5.45								
Female	REF		REF									
Region												
Border							0.70	0.45–1.09	0.69*	0.55–0.87	1.17	0.88–1.54
Border (North)	0.69*	0.56–0.85	0.66*	0.58–0.76	0.88*	0.80–0.98						
Border (Central)	0.44*	0.27–0.72	0.58*	0.44–0.75	1.08	0.91–1.30						
Border (South)	0.55*	0.44–0.69	0.60*	0.52–0.69	0.84*	0.75–0.94						
East TX	0.91	0.82–1.01	0.95	0.90–1.01	0.98	0.91–1.05	0.92	0.69–1.23	0.92	0.80–1.06	0.99	0.80–1.22
Other	REF		REF		REF		REF		REF		REF	
Period												
2006–2010	REF		REF		REF		REF		REF		REF	
2011–2015	1.07	0.98–1.16	1.14*	1.08–1.19	0.91*	0.87–0.96	0.83	0.66–1.05	1.03	0.92–1.15	1.03	0.87–1.21
2016–2019	1.22*	1.09–1.37	1.09*	1.02–1.17	0.85*	0.79–0.91	0.68*	0.49–0.94	1.02	0.87–1.20	0.85	0.68–1.07
Age range												
20–44	0.08*	0.07–0.09	0.04*	0.03–0.04	0.97	0.93–1.01	0.05*	0.03–0.06	0.08*	0.07–0.09	0.05*	0.04–0.06
45–59	0.64*	0.61–0.68	0.63*	0.61–0.65	1.35*	1.29–1.40	0.34*	0.28–0.40	0.46*	0.42–0.50	0.30*	0.27–0.34
60+	REF		REF		REF		REF		REF		REF	
% NH White	1.07*	1.04–1.09	1.08*	1.07–1.10	0.97*	0.96–0.98	0.99	0.93–1.06	1.07*	1.04–1.10	0.96	0.92–1.01
% Poverty	1.03	0.93–1.14	1.02	0.96–1.08	1.09*	1.03–1.16	1.08	0.84–1.39	1.16*	1.02–1.32	1.36*	1.15–1.61
% Smokers	1.07	0.99–1.16	1.07*	1.03–1.12	1.10*	1.05–1.16	1.07	0.88–1.30	1.18*	1.07–1.30	1.22*	1.06–1.41
% Binge drinking	0.90*	0.82–0.99	1.01	0.96–1.07	0.98	0.92–1.04	1.04	0.81–1.34	0.94	0.83–1.07	0.88	0.73–1.06
Chlamydia rate	1.00*	1.00–1.00	1.00	1.00–1.00	1.00*	1.00–1.00	1.00	0.99–1.01	1.00	1.00–1.00	1.00	0.99–1.00
PCP ratio	1.01	0.99–1.02	1.01	1.00–1.02	0.98*	0.97–0.99	0.95*	0.91–0.99	1.01	0.99–1.03	1.02	0.99–1.05
% Obesity	0.98	0.87–1.10	1.05	0.98–1.13	1.14*	1.06–1.23	0.99	0.69–1.41	1.26*	1.07–1.48	1.01	0.80–1.29
% Uninsured	1.24*	1.13–1.36	1.13*	1.07–1.20	1.15*	1.09–1.22	1.03	0.79–1.33	1.05	0.93–1.20	0.89	0.74–1.06
Dentist ratio			1.01	1.00–1.03								

^a^ Zero‐inflated negative binomial model.

* Significant at *p* < 0.05.

With respect to sociodemographic factors, we found that men were less likely than women to be diagnosed with anal cancer (0.63, [0.59–0.66]) but much more likely to be diagnosed with oropharyngeal cancer (5.23, [5.03–5.45]). Younger persons were significantly less likely than those in the 60+ age range to be diagnosed with all cancers, except for cervical cancer. For cervical cancer only, persons in the 45–59 year age range were more likely to be diagnosed than those in the 60+ age range (1.35, [1.29–1.40]). NH Whites were at higher risk of diagnosis with anal, oropharyngeal, and vulvar cancers, and slightly lower risk of diagnosis with cervical cancer (0.97, [0.96–0.98]). Obesity was positively associated with cervical and vulvar cancers. Poverty and smoking were positively associated with cervical, vulvar, and penile cancers. Additionally, smoking was associated with a slightly increased risk of oropharyngeal cancer. Uninsured persons were at a higher risk of developing anal, oropharyngeal, and cervical cancers. In general, there was not a strong association between chlamydia rates and dentist ratios with any of the studied cancers.

## 4 DISCUSSION

To our knowledge, this is the first study to assess differences in HPV‐associated cancers, including but not limited to cervical cancer incidence rates between different regions of TX. Additionally, the combination of three different data sources allowed us to include a more robust set of risk factors in our adjusted model. We found in our unadjusted model that rates of oropharyngeal cancer are higher in East TX than in the rest of TX, and that both cervical and penile cancer rates are higher in parts of the Border than in the rest of TX. In our adjusted model, we found that some of these associations were attenuated after adjusting for additional risk factors. For example, in the adjusted model, we no longer observed an increased oropharyngeal cancer risk in East TX, and instead observed a significant increased risk of oropharyngeal cancer in NH Whites, who make up most of the East TX population. This is consistent with past studies which have found an increased incidence of oropharyngeal cancer in NH Whites.^19^, ^20^, ^21^, ^22^, ^23^ Similarly, after adjustment, we no longer observed increased rates of cervical cancer at the Border, and instead saw positive association with poverty and uninsured rates. Persons without insurance would be less likely to see a doctor and receive screening for cervical cancer. There was also a positive association between penile cancer and poverty, explaining the increased risk of penile cancer at the Border. Past studies of penile cancer risk factors have also found that patients in countries with higher levels of poverty experience higher risk of penile cancer.^24^, ^25^ Poor hygiene is a known risk factor for penile cancer, and people living in poverty live under harsh conditions that may make genital hygiene practices more difficult.^26^ It must also be noted that lower rates of circumcision may also contribute to poor genital hygiene. Past studies have also found an inverse relationship between penile cancer rates and levels of circumcision.^25^, ^26^ The Hispanic men in these studies had both higher penile cancer rates and lower circumcision rates than their NH White and NH Black counterparts. While circumcision rates were not included as a measure in our present study, they certainly could also have contributed to higher penile cancer rates at the Border, which is predominantly Hispanic.

When comparing time periods, we saw significant trends over time in some, but not all the cancers. There was an overall increase in the diagnosis of oropharyngeal cancer over time, which reflects recent changes seen in other studies of HPV‐associated cancer incidence.^10^ We also noticed an increasing time trend in anal cancer diagnosis and decreasing time trend in cervical and vaginal cancer diagnosis, which are also consistent with previous studies.^27^, ^28^, ^29^, ^30^ Decreasing incidence of cervical cancer is promising and is likely attributed to successful screening practices, as well as a potential indication of the effects of HPV vaccination. Increases in oropharyngeal cancer and anal cancer indicate a need to develop more effective screening modalities, especially in high‐risk populations. Currently, there are no Centers for Disease Control and Prevention (CDC)‐recommended screening modalities for oropharyngeal cancer or anal cancer.

For the rarer cancers, our study was limited by low sample size, contributing to unstable time trends. Future work with larger data sources (such as including the entire United States) would potentially lead to clearer time trends. Additionally, our analysis of some behavioral risk factors was limited by the number of missing values present in the data. The County Health Rankings and Roadmaps program sources its data regarding smoking and drinking habits from the Behavioral Risk Factor Surveillance System (BRFSS). Prior to 2016, BRFSS data relied on aggregated landline‐only data for county estimates. Unfortunately, the resulting estimates were not reliable for many counties with smaller samples. Thus, in earlier years of the County Health Rankings data, nearly two‐thirds of the counties did not report reliable smoking or drinking data, causing us to replace those missing data with values from later years instead. This may have led to some inaccuracies, especially in the time trend analysis. While we performed a second time trend analysis excluding smoking and drinking variables and found negligible overall differences from the first analysis, this limitation may impact the generalizability of our study regarding smoking and drinking results. Additionally, our method of combining three data sources and organizing them into 5‐year periods made assumptions regarding population changes, since our denominators for each period were based on the final year of each period. Therefore, we were unable to capture smaller year‐to‐year changes within our analysis. Finally, our method of choosing to divide counties into Border, East Texas, and Other regions was also a limitation of this study. We chose these regions because of their unique racial/ethnic population characteristics. Proportionally, the Border region had a very high Hispanic population, and East TX had a higher NH Black population than TX overall. However, the Other region contained many of Texas's major metropolitan areas such as Dallas‐Fort Worth, Houston, Austin, and San Antonio, which make up a large proportion of the TX population. As a result, the needs of some of the medically underserved areas contained within this larger Other region may not have been well‐represented. Future studies would ideally create more regions to better capture these differences or use TX PHRs for this purpose.

In conclusion, we found that regional differences in HPV‐associated cancer risk were associated with sociodemographic and behavioral risk factors. Elucidating these risk factors, we encourage more specific prevention efforts in communities with higher poverty and uninsured rates, as well as support initiatives for healthy eating and smoking cessation.

## AUTHOR CONTRIBUTIONS

**Thao N. Hoang:** Conceptualization (equal); formal analysis (equal); methodology (equal); writing – original draft (equal); writing – review and editing (equal). **Abbey B. Berenson:** Conceptualization (equal); funding acquisition (equal); methodology (equal); supervision (equal); writing – review and editing (equal). **Yong Shan:** Formal analysis (equal); writing – original draft (equal); writing – review and editing (equal). **Fangjian Guo:** Writing – review and editing (equal). **Victor Adekanmbi:** Writing – review and editing (equal). **Christine Hsu:** Writing – review and editing (equal). **Xiaoying Yu:** Methodology (equal); writing – review and editing (equal). **Yong‐Fang Kuo:** Conceptualization (equal); formal analysis (equal); funding acquisition (equal); methodology (equal); supervision (equal); writing – review and editing (equal).

## FUNDING INFORMATION

This work was supported with funding from The Cancer Prevention and Research Institute of Texas (RP210130) and The Agency for Healthcare Research and Quality (T32HS026133). Dr. Adekanmbi is supported by a research career development award (K12AR084228: Building Interdisciplinary Research Careers in Women's Health Program‐BIRCWH) from the National Institutes of Health/Office of the Director (OD), National Institute of Allergy and Infectious Diseases (NIAID), and Eunice Kennedy Shriver National Institute of Child Health & Human Development (NICHD). Dr. Hsu is supported by a research career development award (K12AR084228‐20S1: Building Interdisciplinary Research Careers in Women's Health Program‐BIRCWH; Berenson, PI) from the National Institute of Arthritis and Musculoskeletal and Skin Diseases of the National Institutes of Health.

The sponsors had no role in the design and conduct of the study; collection, management, analysis, and interpretation of the data; and preparation, review, or approval of the manuscript, and decision to submit the manuscript for publication.

## CONFLICT OF INTEREST STATEMENT

All authors listed on the manuscript have fulfilled the criteria for authorship, reviewed and approved the paper, and attested to the integrity of this paper. We have no conflict of interest.

## ETHICS STATEMENT

This study did not include human subjects and did not require approval from the University of Texas Medical Branch Institutional Review Board.

## Supporting information


Data S1.

## Data Availability

This study analyzed publicly available, deidentified data. Data can be acquired from the Texas Cancer Registry, United States Census Bureau, and the County Health Rankings and Roadmaps Program for 2006‐2019.
