# Supplementary material for: Trends in HPV‐associated cancer incidence in Texas medically underserved regions
Source: Cancer Med. 2024 Aug 27;13(16):e70133. doi: 10.1002/cam4.70133 (PMC11348903; doi:10.1002/cam4.70133)
Supplement: Supplementary file 1 — Data S1. [file CAM4-13-e70133-s001.docx]

**Appendix**

**Supplemental Table 1.** Age-adjusted HPV-associated cancer incidence rates^†^ in Texas regions (2006-2019)

|  | **Year of Diagnosis** | | |
| --- | --- | --- | --- |
|  | **2006-2010** | **2011-2015** | **2016-2019** |
| **Cancer type/region** | **IR (95% CI)** | **IR (95% CI)** | **IR (95% CI)** |
| **Anal** |  |  |  |
| Border | 1.2 (0.97-1.45) | 1.3 (1.04-1.51) | 1.1 (0.88-1.31) |
| East TX | 2.5 (2.13-2.88) | 2.6 (2.21-2.97) | 2.7 (2.36-3.13) |
| Other | 2.0 (1.92-2.14) | 2.1 (2.04-2.25) | 2.1 (1.97-2.17) |
| **Oropharyngeal** |  |  |  |
| Border | 2.9 (2.57-3.31) | 3.1 (2.80-3.54) | 2.8 (2.50-3.19) |
| East TX | 8.0 (7.38-8.73) | 8.7 (8.06-9.45) | 8.2 (7.55-8.88) |
| Other | 5.9 (5.72-6.08) | 6.8 (6.58-6.94) | 6.0 (5.84-6.17) |
| **Cervical^‡^** |  |  |  |
| Border | 13.3 (12.22-14.42) | 15.0 (13.89-16.13) | 12.1 (11.13-13.12) |
| East TX | 12.6 (11.42-13.80) | 11.3 (10.28-12.51) | 9.9 (8.94-11.01) |
| Other | 12.0 (11.66-12.38) | 11.4 (11.02-11.69) | 9.2 (8.92-9.49) |
| **Vaginal^‡^** |  |  |  |
| Border | 0.7 (0.48-0.99) | 0.4 (0.26-0.65) | 0.5 (0.31-0.70) |
| East TX | 0.6 (0.38-0.90) | 1.0 (0.69-1.35) | 0.6 (0.36-0.86) |
| Other | 0.7 (0.59-0.76) | 0.6 (0.52-0.67) | 0.5 (0.41-0.54) |
| **Vulvar^‡^** |  |  |  |
| Border | 1.3 (0.98-1.67) | 1.5 (1.20-1.92) | 1.4 (1.13-1.82) |
| East TX | 2.9 (2.42-3.57) | 3.7 (3.15-4.43) | 2.6 (2.16-3.23) |
| Other | 2.1 (1.99-2.29) | 2.1 (1.99-2.27) | 2.0 (1.84-2.11) |
| **Penile^§^** |  |  |  |
| Border | 1.9 (1.48-2.35) | 1.6 (1.30-2.08) | 1.8 (1.42-2.20) |
| East TX | 1.4 (1.08-1.90) | 1.7 (1.36-2.25) | 0.8 (0.57-1.18) |
| Other | 1.0 (0.92-1.14) | 1.0 (0.94-1.15) | 0.9 (0.77-0.95) |
| **^†^**Per 100,000 persons, age-adjusted to the 2000 US Standard Population  **^‡^**For cervical, vaginal, and vulvar cancers, denominator only includes female population **^§^**For penile cancer, denominator only includes male population | | | |

| **Supplemental Table 2**. Risk of HPV-associated anal cancer by time period, adjusted (2006-2019) | | | | | | | | | | | | |
| --- | --- | --- | --- | --- | --- | --- | --- | --- | --- | --- | --- | --- |
|  | **Year of Diagnosis** | | | | | | | | | | | |
|  | **2006-2010** | |  | | | **2011-2015** | |  | | | **2016-2019**† | |
| **Variable** | **RR** | **(95% CI)** | |  | **RR** | | **(95% CI)** | |  | **RR** | | **(95% CI)** |
| **Region** |  |  | |  |  | |  | |  |  | |  |
| **Border (North)** | 0.59* | (0.40-0.88) | |  | 0.65* | | (0.46-0.91) | |  | 0.75 | | (0.52-1.07) |
| **Border (Central)** | 0.45 | (0.19-1.07) | |  | 0.39* | | (0.16-0.94) | |  | 0.47 | | (0.20-1.06) |
| **Border (South)** | 0.56* | (0.37-0.87) | |  | 0.67 | | (0.45-1.00) | |  | 0.49* | | (0.35-0.70) |
| **East TX** | 0.96 | (0.79-1.17) | |  | 0.88 | | (0.74-1.06) | |  | 0.87 | | (0.73-1.04) |
| **Other** | REF |  | |  | REF | |  | |  | REF | |  |
| **Sex** |  |  | |  |  | |  | |  |  | |  |
| **Male** | 0.68* | (0.62-0.76) | |  | 0.64* | | (0.59-0.71) | |  | 0.57* | | (0.52-0.62) |
| **Female** | REF |  | |  | REF | |  | |  | REF | |  |
| **Age** |  |  | |  |  | |  | |  |  | |  |
| **20-44** | 0.11* | (0.09-0.12) | |  | 0.08* | | (0.07-0.09) | |  | 0.06* | | (0.05-0.07) |
| **45-59** | 0.71* | (0.64-0.79) | |  | 0.66* | | (0.60-0.73) | |  | 0.57* | | (0.52-0.63) |
| **60+** | REF |  | |  | REF | |  | |  | REF | |  |
| **% NH White** | 1.01 | (0.97-1.06) | |  | 1.08* | | (1.03-1.12) | |  | 1.09* | | (1.05-1.13) |
| **% Poverty** | 1.02 | (0.85-1.22) | |  | 0.96 | | (0.79-1.17) | |  | 0.97 | | (0.79-1.18) |
| **% Smokers** | 1.16* | (1.02-1.31) | |  | 1.09 | | (0.97-1.23) | |  | 1.30 | | (0.83-2.04) |
| **% Binge drinking** | 0.81* | (0.69-0.95) | |  | 0.91 | | (0.80-1.05) | |  | 0.82 | | (0.55-1.22) |
| **Chlamydia rate** | 1.00 | (1.00-1.01) | |  | 1.00* | | (1.00-1.01) | |  | 1.00 | | (1.00-1.01) |
| **PCP ratio** | 0.99 | (0.97-1.02) | |  | 1.01 | | (0.98-1.05) | |  | 1.02 | | (0.99-1.05) |
| **% Obesity** | 0.85 | (0.60-1.21) | |  | 0.85 | | (0.69-1.06) | |  | 1.02 | | (0.83-1.26) |
| **% Uninsured** | 1.17* | (1.00-1.35) | |  | 1.34* | | (1.10-1.64) | |  | 1.29* | | (1.04-1.60) |
| *Significant at p<0.05  †Significant at p<0.05 for interaction in the full adjusted model | | | | | | | | | | | | |

| **Supplemental Table 3**. Risk of HPV-associated oropharyngeal cancer by time period, adjusted (2006-2019) | | | | | | | | |
| --- | --- | --- | --- | --- | --- | --- | --- | --- |
|  | **Year of Diagnosis** | | | | | | | |
|  | **2006-2010** | |  | **2011-2015**† | |  | **2016-2019**† | |
| **Variable** | **RR** | **(95% CI)** | | **RR** | **(95% CI)** | | **RR** | **(95% CI)** |
| **Region** |  |  |  |  |  |  |  |  |
| **Border (North)** | 0.69* | (0.54-0.90) | | 0.56* | (0.45-0.70) | | 0.69* | (0.55-0.87) |
| **Border (Central)** | 0.70 | (0.43-1.12) | | 0.43* | (0.26-0.70) | | 0.66 | (0.42-1.02) |
| **Border (South)** | 0.68* | (0.52-0.89) | | 0.65* | (0.51-0.84) | | 0.59* | (0.46-0.75) |
| **East TX** | 1.04 | (0.93-1.15) | | 0.97 | (0.87-1.07) | | 0.86* | (0.78-0.96) |
| **Other** | REF |  |  | REF |  |  | REF |  |
| **Sex** |  |  |  |  |  |  |  |  |
| **Male** | 4.49* | (4.18-4.82) | | 5.34* | (5.00-5.70) | | 5.86* | (5.47-6.29) |
| **Female** | REF |  |  | REF |  |  | REF |  |
| **Age** |  |  |  |  |  |  |  |  |
| **20-44** | 0.05* | (0.04-0.06) | | 0.03* | (0.03-0.04) | | 0.03* | (0.02-0.03) |
| **45-59** | 0.73* | (0.69-0.77) | | 0.62* | (0.59-0.66) | | 0.55* | (0.52-0.58) |
| **60+** | REF |  |  | REF |  |  | REF |  |
| **% NH White** | 1.06* | (1.03-1.09) | | 1.09* | (1.06-1.11) | | 1.09* | (1.07-1.12) |
| **% Poverty** | 0.98 | (0.88-1.08) | | 0.97 | (0.87-1.09) | | 0.96 | (0.85-1.08) |
| **% Smokers** | 1.10* | (1.02-1.18) | | 1.08* | (1.01-1.15) | | 1.41* | (1.08-1.85) |
| **% Binge drinking** | 0.93 | (0.84-1.02) | | 1.02 | (0.94-1.1) | | 0.96 | (0.76-1.21) |
| **Dentist rate** | 1.01 | (0.98-1.04) | | 1.01 | (0.99-1.04) | | 1.02 | (0.99-1.04) |
| **Chlamydia rate** | 1.00 | (1.00-1.00) | | 1.00* | (1.00-1.00) | | 1.00 | (1.00-1.00) |
| **PCP ratio** | 1.01 | (1.00-1.03) | | 1.00 | (0.98-1.02) | | 1.00 | (0.98-1.02) |
| **% Obesity** | 1.13 | (0.92-1.38) | | 0.84* | (0.74-0.94) | | 1.10 | (0.97-1.25) |
| **% Uninsured** | 1.14* | (1.05-1.25) | | 1.21* | (1.08-1.36) | | 1.09 | (0.96-1.24) |
| *Significant at p<0.05  †Significant at p<0.05 for interaction in the full adjusted model | | | | | | | | |

| **Supplemental Table 4**. Risk of HPV-associated cervical cancer by time period, adjusted (2006-2019) | | | | | | | | | |
| --- | --- | --- | --- | --- | --- | --- | --- | --- | --- |
|  | **Year of Diagnosis** | | | | | | | | |
|  | **2006-2010** | |  | **2011-2015**† | |  | | **2016-2019**† | |
| **Variable** | **RR** | **(95% CI)** | | **RR** | **(95% CI)** | | **RR** | | **(95% CI)** |
| **Region** |  |  |  |  |  |  | |  |  |
| **Border (North)** | 0.78* | (0.64-0.95) |  | 0.91 | (0.77-1.07) | | 0.90 | | (0.74-1.09) |
| **Border (Central)** | 0.90 | (0.64-1.27) |  | 1.27 | (0.96-1.67) | | 0.96 | | (0.69-1.33) |
| **Border (South)** | 0.76* | (0.61-0.94) |  | 0.95 | (0.77-1.18) | | 0.78* | | (0.65-0.94) |
| **East TX** | 1.01 | (0.90-1.14) |  | 0.98 | (0.87-1.11) | | 0.91 | | (0.80-1.04) |
| **Other** | REF |  |  | REF |  |  | | REF |  |
| **Age** |  |  |  |  |  |  | |  |  |
| **20-44** | 0.90* | (0.84-0.97) |  | 0.91* | (0.85-0.98) | | 1.11* | | (1.03-1.19) |
| **45-59** | 1.26* | (1.17-1.35) |  | 1.31* | (1.22-1.41) | | 1.48* | | (1.37-1.60) |
| **60+** | REF |  |  | REF |  |  | | REF |  |
| **% NH White** | 0.95* | (0.93-0.98) |  | 0.97* | (0.95-1.00) | | 0.99 | | (0.96-1.02) |
| **% Poverty** | 1.12* | (1.02-1.24) |  | 0.98 | (0.87-1.11) | | 1.18* | | (1.04-1.34) |
| **% Smokers** | 1.17* | (1.08-1.26) |  | 1.05 | (0.97-1.13) | | 1.12 | | (0.83-1.50) |
| **% Binge drinking** | 0.95 | (0.86-1.05) |  | 1.01 | (0.93-1.10) | | 0.95 | | (0.72-1.25) |
| **Chlamydia rate** | 1.00 | (1.0-1.0) |  | 1.00* | (1.00-1.01) | | 1.00 | | (1.00-1.00) |
| **PCP ratio** | 1.00 | (0.98-1.01) |  | 0.97* | (0.94-0.99) | | 0.96* | | (0.94-0.98) |
| **% Obesity** | 1.15 | (0.95-1.39) |  | 1.08 | (0.95-1.23) | | 1.12 | | (0.98-1.29) |
| **% Uninsured** | 1.07 | (0.99-1.17) |  | 1.22* | (1.08-1.37) | | 1.15* | | (1.00-1.33) |
| *Significant at p<0.05  †Significant at p<0.05 for interaction in the full adjusted model | | | | | | | | | |

| **Supplemental Table 5**. Risk of HPV-associated vaginal cancer by time period, adjusted (2006-2019) | | | | | | | | |
| --- | --- | --- | --- | --- | --- | --- | --- | --- |
|  | **Year of Diagnosis** | | | | | | | |
|  | **2006-2010** | |  | **2011-2015** | |  | **2016-2019**† | |
| **Variable** | **RR** | **(95% CI)** | | **RR** | **(95% CI)** | | **RR** | **(95% CI)** |
| **Region** |  |  |  |  |  |  |  |  |
| **Border** | 0.63 | (0.29-1.37) | | 0.57 | (0.27-1.20) | | 1.07 | (0.05-2.31) |
| **East TX** | 0.61 | (0.36-1.03) | | 1.22 | (0.78-1.91) | | 1.01 | (0.58-1.75) |
| **Other** | REF | |  | REF | |  | REF |  |
| **Age** |  |  |  |  |  |  |  |  |
| **20-44** | 0.05* | (0.03-0.09) | | 0.05* | (0.03-0.08) | | 0.04* | (0.02-0.07) |
| **45-59** | 0.31* | (0.22-0.42) | | 0.36* | (0.27-0.49) | | 0.37* | (0.27-0.51) |
| **60+** | REF | |  | REF | |  | REF |  |
| **% NH White** | 0.98 | (0.87-1.11) | | 1.00 | (0.88-1.13) | | 0.96 | (0.84-1.08) |
| **% Poverty** | 1.17 | (0.82-1.66) | | 1.32 | (0.84-2.07) | | 0.76 | (0.42-1.38) |
| **% Smokers** | 0.94 | (0.68-1.28) | | 1.15 | (0.87-1.52) | | 4.36* | (1.02-18.67) |
| **% Binge drinking** | 0.95 | (0.64-1.41) | | 1.06 | (0.75-1.50) | | 1.08 | (0.34-3.5) |
| **Chlamydia rate** | 1.00 | (0.99-1.01) | | 1.00 | (0.99-1.01) | | 1.00 | (0.99-1.01) |
| **PCP ratio** | 0.92* | (0.86-0.97) | | 0.98 | (0.90-1.07) | | 0.96 | (0.88-1.06) |
| **% Obesity**^§^ |  |  |  | 1.15 | (0.66-1.99) | | 0.72 | (0.39-1.33) |
| **% Uninsured**^§^ |  |  |  | 0.83 | (0.47-1.46) | | 0.81 | (0.39-1.66) |
| *Significant at p<0.05, †Significant at p<0.05 for interaction in the full adjusted model, §Could not be added for 2006-2010 due to converging issues | | | | | | | | |

| **Supplemental Table 6**. Risk of HPV-associated vulvar cancer by time period, adjusted (2006-2019) | | | | | | | | |
| --- | --- | --- | --- | --- | --- | --- | --- | --- |
|  | **Year of Diagnosis** | | | | | | | |
|  | **2006-2010** | |  | **2011-2015** | |  | **2016-2019** | |
| **Variable** | **RR** | **(95% CI)** | | **RR** | **(95% CI)** | | **RR** | **(95% CI)** |
| **Region** |  |  |  |  |  |  |  |  |
| **Border** | 0.47* | (0.29-0.74) | | 0.87 | (0.61-1.24) | | 0.65* | (0.45-0.94) |
| **East TX** | 0.92 | (0.72-1.18) | | 1.22 | (0.97-1.53) | | 0.66* | (0.52-0.85) |
| **Other** | REF |  |  | REF |  |  | REF |  |
| **Age** |  |  |  |  |  |  |  |  |
| **20-44** | 0.10* | (0.08-0.12) | | 0.08* | (0.06-0.09) | | 0.07* | (0.05-0.09) |
| **45-59** | 0.46* | (0.40-0.53) | | 0.48* | (0.42-0.54) | | 0.44* | (0.39-0.51) |
| **60+** | REF |  |  | REF |  |  | REF |  |
| **% NH White** | 1.06 | (1.00-1.13) | | 1.04 | (0.98-1.11) | | 1.06* | (1.00-1.12) |
| **% Poverty** | 1.46* | (1.17-1.82) | | 0.94 | (0.74-1.20) | | 1.01 | (0.77-1.32) |
| **% Smokers** | 1.20* | (1.02-1.42) | | 1.14 | (0.98-1.32) | | 3.29* | (1.81-5.98) |
| **% Binge drinking** | 0.93 | (0.75-1.15) | | 0.93 | (0.77-1.11) | | 0.69 | (0.39-1.22) |
| **Chlamydia rate** | 1.00 | (0.99-1.00) | | 1.00 | (1.00-1.01) | | 1.00 | (0.99-1.00) |
| **PCP ratio** | 1.03* | (1.00-1.07) | | 0.97 | (0.93-1.02) | | 0.99 | (0.95-1.03) |
| **% Obesity** | 0.83 | (0.54-1.28) | | 1.09 | (0.82-1.44) | | 1.19 | (0.89-1.60) |
| **% Uninsured** | 0.99 | (0.81-1.21) | | 1.02 | (0.78-1.34) | | 0.93 | (0.69-1.25) |
| *Significant at p<0.05 | | | | | | | |  |

| **Supplemental Table 7**. Risk of HPV-associated penile cancer by time period, adjusted (2006-2019) | | | | | | | | |
| --- | --- | --- | --- | --- | --- | --- | --- | --- |
|  | **Year of Diagnosis** | | | | | | | |
|  | **2006-2010** | |  | **2011-2015** | |  | **2016-2019** | |
| **Variable** | **RR** | **(95% CI)** | | **RR** | **(95% CI)** | | **RR** | **(95% CI)** |
| **Region** |  |  |  |  |  |  |  |  |
| **Border** | 0.70 | (0.39-1.26) | | 1.40 | (0.88-2.23) | | 1.25 | (0.77-2.02) |
| **East TX** | 1.02 | (0.71-1.47) | | 1.23 | (0.88-1.70) | | 0.66 | (0.43-1.03) |
| **Other** | REF | |  | REF |  |  | REF |  |
| **Age** |  |  |  |  |  |  |  |  |
| **20-44** | 0.06* | (0.04-0.08) | | 0.05* | (0.04-0.07) | | 0.04* | (0.03-0.06) |
| **45-59** | 0.26* | (0.21-0.32) | | 0.35* | (0.28-0.43) | | 0.29* | (0.23-0.36) |
| **60+** | REF | |  | REF |  |  | REF |  |
| **% NH White** | 0.93 | (0.84-1.02) | | 1.01 | (0.93-1.10) | | 0.94 | (0.86-1.03) |
| **% Poverty** | 1.62* | (1.19-2.19) | | 1.38* | (1.02-1.87) | | 1.11 | (0.75-1.67) |
| **% Smokers** | 1.13 | (0.87-1.45) | | 1.19 | (0.96-1.48) | | 1.77 | (0.71-4.39) |
| **% Binge drinking** | 0.77 | (0.56-1.07) | | 0.99 | (0.76-1.28) | | 1.37 | (0.57-3.32) |
| **Chlamydia rate** | 1.00 | (0.99-1.01) | | 1.00 | (1.00-1.01) | | 1.00 | (0.99-1.00) |
| **PCP ratio** | 1.01 | (0.97-1.06) | | 1.05 | (0.98-1.11) | | 0.99 | (0.92-1.06) |
| **% Obesity** | 0.87 | (0.47-1.63) | | 0.91 | (0.63-1.31) | | 1.22 | (0.78-1.88) |
| **% Uninsured** | 0.83 | (0.62-1.12) | | 0.89 | (0.61-1.30) | | 1.09 | (0.70-1.70) |
| *Significant at p<0.05 | | | | | | | |  |

| **Supplemental Table 8**. Risk of HPV-associated anal cancer by time period, adjusted, excluding smoking and drinking (2006-2019) | | | | | | | | | | | | |
| --- | --- | --- | --- | --- | --- | --- | --- | --- | --- | --- | --- | --- |
|  | **Year of Diagnosis** | | | | | | | | | | | |
|  | **2006-2010** | |  | | | **2011-2015** | |  | | | **2016-2019**† | |
| **Variable** | **RR** | **(95% CI)** | |  | **RR** | | **(95% CI)** | |  | **RR** | | **(95% CI)** |
| **Region** |  |  | |  |  | |  | |  |  | |  |
| **Border (North)** | 0.63* | (0.42-0.93) | |  | 0.65* | | (0.46-0.92) | |  | 0.74 | | (0.52-1.06) |
| **Border (Central)** | 0.45 | (0.19-1.06) | |  | 0.39* | | (0.16-0.95) | |  | 0.49 | | (0.22-1.12) |
| **Border (South)** | 0.55* | (0.36-0.86) | |  | 0.64* | | (0.44-0.95) | |  | 0.49* | | (0.35-0.70) |
| **East TX** | 1.02 | (0.84-1.23) | |  | 0.91 | | (0.76-1.09) | |  | 0.88 | | (0.74-1.06) |
| **Other** | REF |  | |  | REF | |  | |  | REF | |  |
| **Sex** |  |  | |  |  | |  | |  |  | |  |
| **Male** | 0.68* | (0.62-0.76) | |  | 0.64* | | (0.59-0.71) | |  | 0.57* | | (0.52-0.62) |
| **Female** | REF |  | |  | REF | |  | |  | REF | |  |
| **Age** |  |  | |  |  | |  | |  |  | |  |
| **20-44** | 0.11* | (0.09-0.12) | |  | 0.08* | | (0.07-0.09) | |  | 0.06* | | (0.05-0.07) |
| **45-59** | 0.71* | (0.64-0.79) | |  | 0.66* | | (0.60-0.73) | |  | 0.57* | | (0.52-0.63) |
| **60+** | REF |  | |  | REF | |  | |  | REF | |  |
| **% NH White** | 1.03 | (0.99-1.08) | |  | 1.09* | | (1.05-1.14) | |  | 1.10* | | (1.06-1.13) |
| **% Poverty** | 1.04 | (0.88-1.24) | |  | 0.99 | | (0.81-1.20) | |  | 1.02 | | (0.85-1.22) |
| **Chlamydia rate** | 1.00 | (1.00-1.01) | |  | 1.01* | | (1.00-1.01) | |  | 1.00 | | (1.00-1.01) |
| **PCP ratio** | 0.99 | (0.97-1.01) | |  | 1.01 | | (0.98-1.05) | |  | 1.02 | | (0.99-1.05) |
| **% Obesity** | 0.97 | (0.70-1.36) | |  | 0.90 | | (0.74-1.11) | |  | 1.12 | | (0.94-1.32) |
| **% Uninsured** | 1.15 | (0.99-1.33) | |  | 1.37* | | (1.12-1.67) | |  | 1.36* | | (1.12-1.66) |
| *Significant at p<0.05  †Significant at p<0.05 for interaction in the full adjusted model | | | | | | | | | | | | |

| **Supplemental Table 9**. Risk of HPV-associated oropharyngeal cancer by time period, adjusted, excluding smoking and drinking (2006-2019) | | | | | | | | |
| --- | --- | --- | --- | --- | --- | --- | --- | --- |
|  | **Year of Diagnosis** | | | | | | | |
|  | **2006-2010** | |  | **2011-2015**† | |  | **2016-2019**† | |
| **Variable** | **RR** | **(95% CI)** | | **RR** | **(95% CI)** | | **RR** | **(95% CI)** |
| **Region** |  |  |  |  |  |  |  |  |
| **Border (North)** | 0.69* | (0.54-0.89) | | 0.54* | (0.44-0.67) | | 0.68* | (0.55-0.85) |
| **Border (Central)** | 0.68 | (0.42-1.08) | | 0.40* | (0.26-0.66) | | 0.66 | (0.43-1.02) |
| **Border (South)** | 0.64* | (0.50-0.83) | | 0.60* | (0.48-0.76) | | 0.57* | (0.46-0.71) |
| **East TX** | 1.06 | (0.95-1.18) | | 0.98 | (0.88-1.08) | | 0.88* | (0.79-0.97) |
| **Other** | REF |  |  | REF |  |  | REF |  |
| **Sex** |  |  |  |  |  |  |  |  |
| **Male** | 4.48* | (4.18-4.81) | | 5.33* | (4.99-5.69) | | 5.84* | (5.45-6.26) |
| **Female** | REF |  |  | REF |  |  | REF |  |
| **Age** |  |  |  |  |  |  |  |  |
| **20-44** | 0.05* | (0.04-0.06) | | 0.03* | (0.03-0.04) | | 0.03* | (0.02-0.03) |
| **45-59** | 0.73* | (0.69-0.77) | | 0.63* | (0.59-0.66) | | 0.55* | (0.53-0.59) |
| **60+** | REF |  |  | REF |  |  | REF |  |
| **% NH White** | 1.07* | (1.04-1.10) | | 1.09* | (1.07-1.11) | | 1.10* | (1.08-1.13) |
| **% Poverty** | 0.99 | (0.89-1.09) | | 0.98 | (0.87-1.09) | | 1.02 | (0.92-1.13) |
| **Dentist rate** | 1.01 | (0.98-1.04) | | 1.01 | (0.99-1.04) | | 1.02 | (0.99-1.04) |
| **Chlamydia rate** | 1.00 | (1.00-1.00) | | 1.00 | (1.00-1.00) | | 1.00 | (1.00-1.00) |
| **PCP ratio** | 1.01* | (1.00-1.03) | | 1.00 | (0.98-1.02) | | 1.00 | (0.99-1.02) |
| **% Obesity** | 1.19 | (0.99-1.43) | | 0.86* | (0.77-0.96) | | 1.17* | (1.06-1.28) |
| **% Uninsured** | 1.14* | (1.04-1.24) | | 1.24* | (1.11-1.39) | | 1.11 | (0.99-1.24) |
| *Significant at p<0.05  †Significant at p<0.05 for interaction in the full adjusted model | | | | | | | | |

| **Supplemental Table 10**. Risk of HPV-associated cervical cancer by time period, adjusted, excluding smoking and drinking (2006-2019) | | | | | | | | | |
| --- | --- | --- | --- | --- | --- | --- | --- | --- | --- |
|  | **Year of Diagnosis** | | | | | | | | |
|  | **2006-2010** | |  | **2011-2015**† | |  | | **2016-2019**† | |
| **Variable** | **RR** | **(95% CI)** | | **RR** | **(95% CI)** | | **RR** | | **(95% CI)** |
| **Region** |  |  |  |  |  |  | |  |  |
| **Border (North)** | 0.81* | (0.67-0.99) |  | 0.91 | (0.77-1.07) | | 0.90 | | (0.75-1.08) |
| **Border (Central)** | 0.90 | (0.64-1.27) |  | 1.24 | (0.95-1.63) | | 0.98 | | (0.71-1.35) |
| **Border (South)** | 0.74* | (0.60-0.92) |  | 0.93 | (0.75-1.14) | | 0.78* | | (0.65-0.94) |
| **East TX** | 1.04 | (0.93-1.17) |  | 0.99 | (0.88-1.12) | | 0.91 | | (0.80-1.04) |
| **Other** | REF |  |  | REF |  |  | | REF |  |
| **Age** |  |  |  |  |  |  | |  |  |
| **20-44** | 0.90* | (0.84-0.97) |  | 0.91* | (0.85-0.98) | | 1.11* | | (1.03-1.19) |
| **45-59** | 1.26* | (1.17-1.35) |  | 1.31* | (1.22-1.41) | | 1.48* | | (1.37-1.60) |
| **60+** | REF |  |  | REF |  |  | | REF |  |
| **% NH White** | 0.97* | (0.95-1.00) |  | 0.98* | (0.95-1.00) | | 0.99 | | (0.97-1.02) |
| **% Poverty** | 1.14* | (1.04-1.26) |  | 0.99 | (0.88-1.12) | | 1.20* | | (1.07-1.35) |
| **Chlamydia rate** | 1.00 | (1.00-1.00) |  | 1.00* | (1.00-1.01) | | 1.00 | | (1.00-1.00) |
| **PCP ratio** | 0.99 | (0.98-1.01) |  | 0.96* | (0.94-0.99) | | 0.96* | | (0.94-0.98) |
| **% Obesity** | 1.26* | (1.05-1.52) |  | 1.10 | (0.97-1.25) | | 1.16* | | (1.04-1.30) |
| **% Uninsured** | 1.08 | (0.99-1.18) |  | 1.22* | (1.08-1.37) | | 1.17* | | (1.02-1.33) |
| *Significant at p<0.05  †Significant at p<0.05 for interaction in the full adjusted model | | | | | | | | | |

| **Supplemental Table 11**. Risk of HPV-associated vaginal cancer by time period, adjusted, excluding smoking and drinking (2006-2019) | | | | | | | | |
| --- | --- | --- | --- | --- | --- | --- | --- | --- |
|  | **Year of Diagnosis** | | | | | | | |
|  | **2006-2010** | |  | **2011-2015** | |  | **2016-2019**† | |
| **Variable** | **RR** | **(95% CI)** | | **RR** | **(95% CI)** | | **RR** | **(95% CI)** |
| **Region** |  |  |  |  |  |  |  |  |
| **Border** | 0.71 | (0.32-1.57) | | 0.55 | (0.27-1.13) | | 1.03 | (0.48-2.23) |
| **East TX** | 0.63 | (0.37-1.06) | | 1.24 | (0.80-1.91) | | 0.96 | (0.55-1.69) |
| **Other** | REF | |  | REF | |  | REF |  |
| **Age** |  |  |  |  |  |  |  |  |
| **20-44** | 0.05* | (0.03-0.09) | | 0.05* | (0.03-0.08) | | 0.03* | (0.02-0.06) |
| **45-59** | 0.31* | (0.23-0.41) | | 0.36* | (0.27-0.49) | | 0.35* | (0.26-0.49) |
| **60+** | REF | |  | REF | |  | REF |  |
| **% NH White** | 0.99 | (0.89-1.11) | | 1.01 | (0.91-1.14) | | 0.99 | (0.89-1.11) |
| **% Poverty** | 1.04 | (0.70-1.54) | | 1.33 | (0.85-2.07) | | 0.98 | (0.58-1.66) |
| **Chlamydia rate** | 1.00 | (0.99-1.01) | | 1.00 | (0.99-1.01) | | 1.00 | (0.99-1.01) |
| **PCP ratio** | 0.92* | (0.87-0.98) | | 0.97 | (0.89-1.06) | | 0.95 | (0.87-1.04) |
| **% Obesity**^§^ |  |  |  | 1.19 | (0.69-2.04) | | 0.86 | (0.50-1.48) |
| **% Uninsured**^§^ |  |  |  | 0.86 | (0.49-1.52) | | 0.85 | (0.46-1.57) |
| *Significant at p<0.05, †Significant at p<0.05 for interaction in the full adjusted model, §Could not be added for 2006-2010 due to converging issues | | | | | | | | |

| **Supplemental Table 12**. Risk of HPV-associated vulvar cancer by time period, adjusted, excluding smoking and drinking (2006-2019) | | | | | | | | |
| --- | --- | --- | --- | --- | --- | --- | --- | --- |
|  | **Year of Diagnosis** | | | | | | | |
|  | **2006-2010** | |  | **2011-2015** | |  | **2016-2019** | |
| **Variable** | **RR** | **(95% CI)** | | **RR** | **(95% CI)** | | **RR** | **(95% CI)** |
| **Region** |  |  |  |  |  |  |  |  |
| **Border** | 0.46* | (0.29-0.74) | | 0.85 | (0.60-1.21) | | 0.67* | (0.46-0.96) |
| **East TX** | 0.96 | (0.75-1.22) | | 1.27* | (1.01-1.58) | | 0.68* | (0.53-0.88) |
| **Other** | REF |  |  | REF |  |  | REF |  |
| **Age** |  |  |  |  |  |  |  |  |
| **20-44** | 0.10* | (0.08-0.12) | | 0.08* | (0.06-0.09) | | 0.07* | (0.05-0.09) |
| **45-59** | 0.46* | (0.40-0.53) | | 0.47* | (0.41-0.54) | | 0.44* | (0.39-0.51) |
| **60+** | REF |  |  | REF |  |  | REF |  |
| **% NH White** | 1.09* | (1.03-1.15) | | 1.06 | (1.01-1.12) | | 1.10* | (1.05-1.16) |
| **% Poverty** | 1.49* | (1.20-1.85) | | 0.96 | (0.76-1.22) | | 1.27* | (1.00-1.61) |
| **Chlamydia rate** | 1.00 | (0.99-1.00) | | 1.00 | (1.00-1.01) | | 1.00 | (0.99-1.00) |
| **PCP ratio** | 1.03* | (1.00-1.06) | | 0.97 | (0.93-1.01) | | 0.99 | (0.95-1.03) |
| **% Obesity** | 0.93 | (0.61-1.41) | | 1.14 | (0.87-1.50) | | 1.62* | (1.27-2.05) |
| **% Uninsured** | 0.98 | (0.80-1.20) | | 1.05 | (0.80-1.37) | | 1.04 | (0.80-1.36) |
| *Significant at p<0.05 | | | | | | | |  |

| **Supplemental Table 13**. Risk of HPV-associated penile cancer by time period, adjusted, excluding smoking and drinking (2006-2019) | | | | | | | | |
| --- | --- | --- | --- | --- | --- | --- | --- | --- |
|  | **Year of Diagnosis** | | | | | | | |
|  | **2006-2010** | |  | **2011-2015** | |  | **2016-2019** | |
| **Variable** | **RR** | **(95% CI)** | | **RR** | **(95% CI)** | | **RR** | **(95% CI)** |
| **Region** |  |  |  |  |  |  |  |  |
| **Border** | 0.74 | (0.41-1.32) | | 1.35 | (0.85-2.15) | | 1.31 | (0.81-2.11) |
| **East TX** | 1.10 | (0.77-1.57) | | 1.28 | (0.93-1.77) | | 0.66 | (0.42-1.02) |
| **Other** | REF | |  | REF |  |  | REF |  |
| **Age** |  |  |  |  |  |  |  |  |
| **20-44** | 0.06* | (0.04-0.08) | | 0.05* | (0.04-0.07) | | 0.04* | (0.03-0.06) |
| **45-59** | 0.26* | (0.21-0.32) | | 0.34* | (0.28-0.42) | | 0.29* | (0.23-0.37) |
| **60+** | REF | |  | REF |  |  | REF |  |
| **% NH White** | 0.95 | (0.87-1.03) | | 1.01 | (0.95-1.12) | | 0.97 | (0.89-1.05) |
| **% Poverty** | 1.62* | (1.19-2.19) | | 1.40* | (1.04-1.89) | | 1.19 | (0.83-1.71) |
| **Chlamydia rate** | 1.00 | (0.99-1.01) | | 1.00 | (1.00-1.01) | | 1.00 | (0.99-1.00) |
| **PCP ratio** | 1.01 | (0.96-1.06) | | 1.03 | (0.97-1.10) | | 0.98 | (0.91-1.04) |
| **% Obesity** | 0.99 | (0.54-1.80) | | 0.95 | (0.66-1.37) | | 1.32 | (0.91-1.93) |
| **% Uninsured** | 0.83 | (0.62-1.12) | | 0.92 | (0.63-1.34) | | 1.01 | (0.68-1.50) |
| *Significant at p<0.05 | | | | | | | |  |
